# Combined Microscopic Tubular and Endoscopic Approach for Calcified Midline Thoracic Disc Herniation: A Bridge to Endoscopic Discectomy

**DOI:** 10.7759/cureus.66948

**Published:** 2024-08-15

**Authors:** Robert Ziechmann, Sami M Pathak, Bong-Soo Kim

**Affiliations:** 1 Neurosurgery, Temple University Hospital, Philadelphia, USA

**Keywords:** endoscopic discectomy, endoscopic learning curve, microscopic discectomy, minimally invasive spine surgery, thoracic decompression, thoracic disc herniation, thoracic myelopathy

## Abstract

Symptomatic thoracic disc herniation (TDH) is relatively uncommon and can present with thoracolumbar pain, myelopathy, bladder dysfunction, and motor dysfunction. Midline TDHs and calcified discs are more challenging to access and treat compared to the cervical or lumbar region due to the narrow working corridor around the lungs, ribs, and thoracic spinal cord. Open approaches such as the transthoracic or retropleural approach are particularly morbid. Minimally invasive endoscopic techniques offer decreased tissue dissection and manipulation of the thecal sac but involve a more difficult learning curve. We present a posterolateral approach using a minimally invasive tubular retractor and microscope, which is like minimally invasive techniques many surgeons are already accustomed to using, combined with an endoscope through the tubular retractor. The patient is a 57-year-old female who presented with gait instability due to balance problems and mild bilateral leg “heaviness” and weakness. Her neurologic exam was remarkable for bilateral leg weakness, decreased sensation at the T12 level, hyperreflexia in the bilateral lower extremities, a positive Romberg test, and a wide-based gait. Magnetic resonance imaging revealed disc extrusion at T11-T12 and ligamentum flavum infolding causing mild central canal narrowing, resulting in a mass effect on the cord. We performed a minimally invasive discectomy using a tubular approach combined with an endoscope to access the ventral midline without manipulation of the spinal cord. A combined microscopic and endoscopic may allow surgeons already comfortable with microscopic surgery to master the learning curve of endoscopic techniques.

## Introduction

Symptomatic thoracic disc herniation (TDH) accounts for less than 5% of disc herniations [1]. The symptoms of TDH include thoracolumbar pain, myelopathy, bladder dysfunction, sensory deficits, and motor dysfunction [2]. The T11-T12 disc level in particular is vulnerable to degeneration because of high mobility and relatively large posterior longitudinal ligament [1].

Midline TDHs and calcified discs are more challenging to access and treat than the more common cervical or lumbar disc herniation [3,4]. This is because of the added difficulty of the narrow working corridor around the lungs, the ribs, and the thoracic spinal cord [3,4]. They usually require a transthoracic or retropleural approach, both of which may be particularly morbid, compared to the posterolateral approach [3,4]. Often, the posterolateral approach is reserved for paracentral and soft discs [4].

Thoracic endoscopic spine surgery may decrease the need for tissue dissection and retraction or manipulation of the thoracic spinal cord/thecal sac [5]. However, endoscopic approaches involve a difficult learning curve [5]. In this article, we present a posterolateral approach using both a minimally invasive tubular retractor and the microscope and adding midline access via an endoscope through the tubular retractor.

## Case presentation

The patient is a 57-year-old female who presented with gait instability due to balance problems and mild bilateral leg “heaviness” and weakness. She has a prior history of lumbar spondylosis and L2-L5 lumbar laminectomy for radicular pain. The patient did well initially following the operative intervention but then developed a return of her preoperative symptoms. She attempted eight weeks of physical therapy but did not demonstrate any improvement in her symptoms. She reported pain at 10/10 that originates in the lower back and radiates down the back of the left leg, stopping behind the left knee, along with left leg "heaviness” and paresthesias. She continued to use a cane for assistance with ambulation and was unable to climb stairs. Her pain was reported as worsening with prolonged sitting, standing, and walking, and she had been unable to ambulate more than one city block. The patient denied bowel and bladder dysfunction or any episodes of incontinence.

Her neurologic examination included: bilateral leg weakness to 4/5; decreased sensation in the T12 sensory level; 3+ reflexes in the bilateral lower extremities; positive Romberg test; and wide-based gait. Laboratory findings were unremarkable except for those demonstrated in Table 1.

**Table 1:** Preoperative laboratory measurements Demonstrated are the relevant abnormal preoperative laboratory values. TSH: thyroid stimulating hormone, HbA1c: hemoglobin A1c

Parameter (units of measurement)	Measured value	Reference range
TSH (mIU/L)	8.29	0.40-4.50
HbA1c (%)	6.9	4.7-6.4

**Figure 1:**
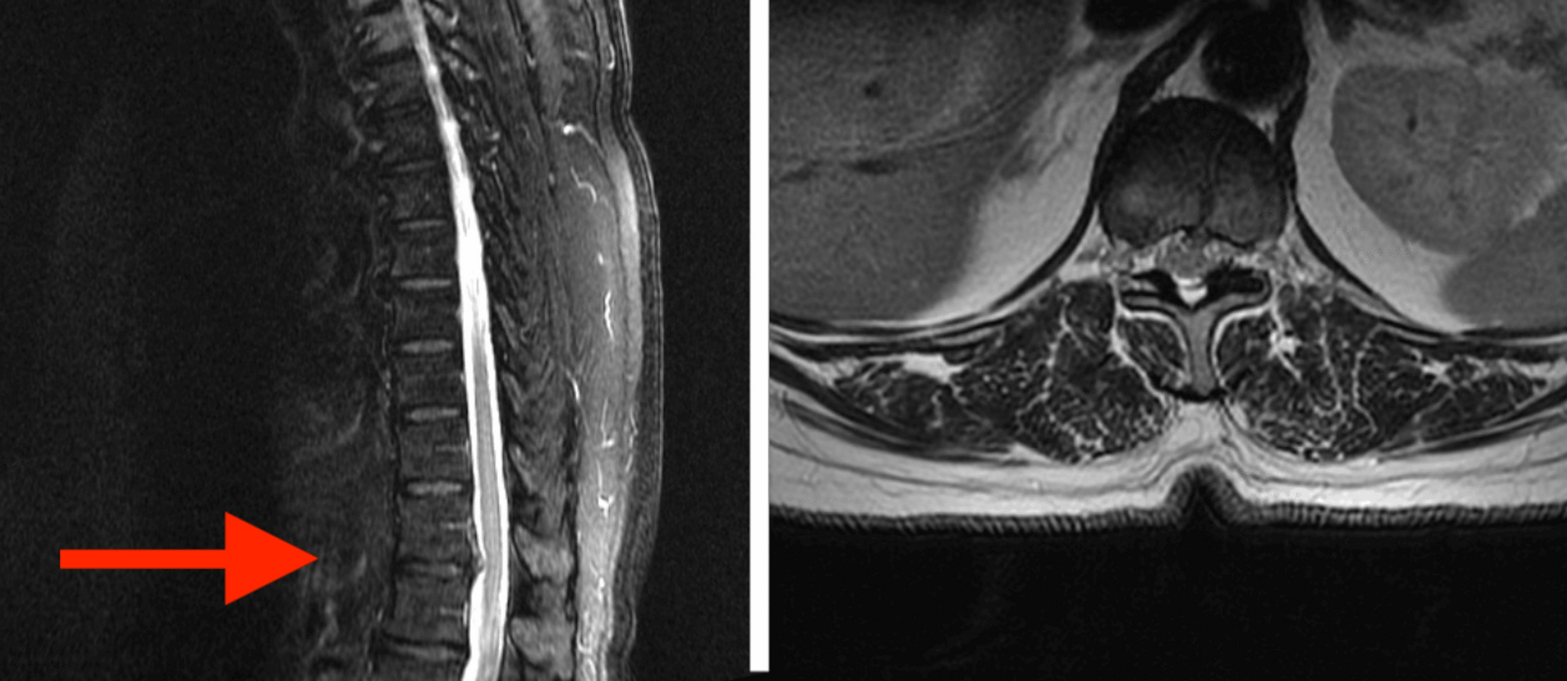
Preoperative imaging demonstrating thoracic disc herniation Preoperative magnetic resonance imaging (MRI) in the sagittal (A) and axial (B) planes revealing disc extrusion at T11-T12 combined with ligamentum flavum infolding causing mild central canal narrowing and resulting in a mass effect on the cord. The arrow in (A) indicates the T11-T12 level.

Magnetic resonance imaging (MRI) revealed disc extrusion at T11-T12 combined with ligamentum flavum infolding causing mild central canal narrowing, resulting in a mass effect on the cord (Figure 1). Given the cord signal change and signs of myelopathy, surgical decompression was necessary. A minimally invasive tubular approach combined with an endoscope allowed for access to the ventral midline without manipulation of the spinal cord.

**Figure 2:**
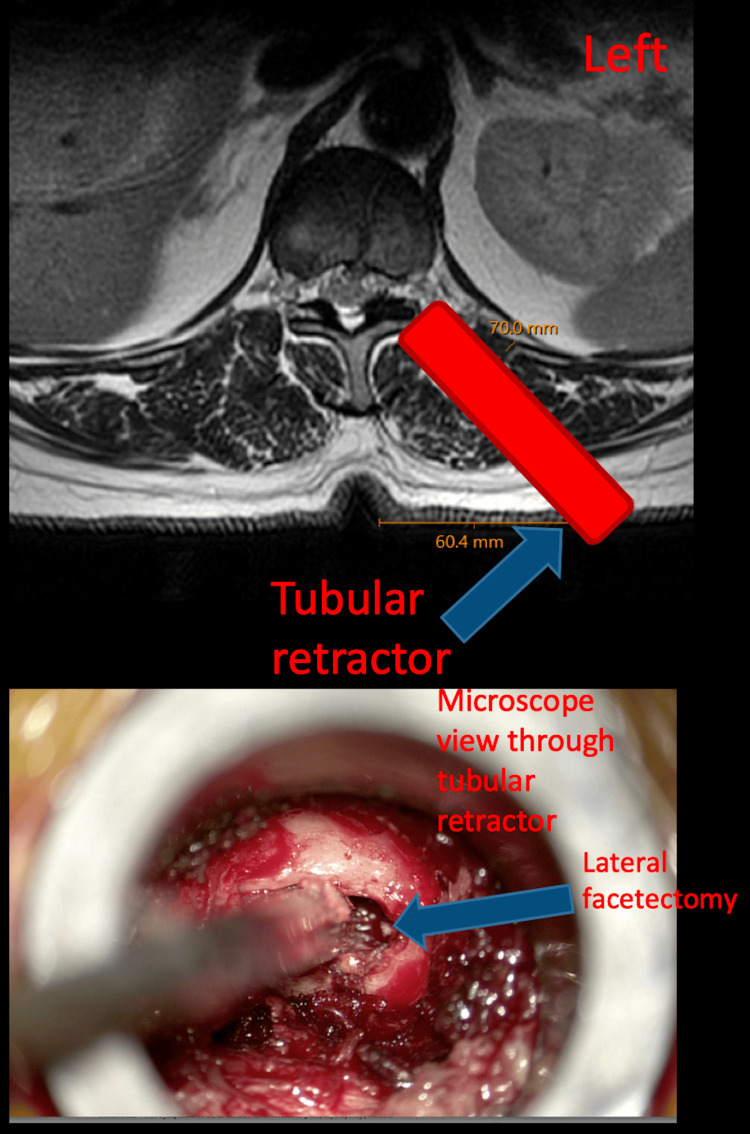
Viewpoint from the microscope Demonstration of lateral facetectomy under the microscope, seen through the tubular retractor.

The patient was positioned prone on a flat Jackson table. Fluoroscopy was used to localize the intended level of surgery. Levels were confirmed by counting from the sacrum. A left-sided paramedian incision was made 5 cm from the midline in the thoracic region. Serial dilators were used to dock the minimally invasive access tube over the medial facet joint and lamina at the level of T11-T12 disc space, and fluoroscopy was used to confirm the correct level. The operative microscope was brought to the field, the soft tissue was dissected, and a medial joint-preserving facetectomy was performed with the high-speed drill (Figure 2). Ligamentum flavum was dissected and removed with the Kerrison. The dura was dissected from the disc annulus with an angled curette. A lateral annulotomy was created with a 15-blade and then expanded using a pituitary rongeur.

**Figure 3:**
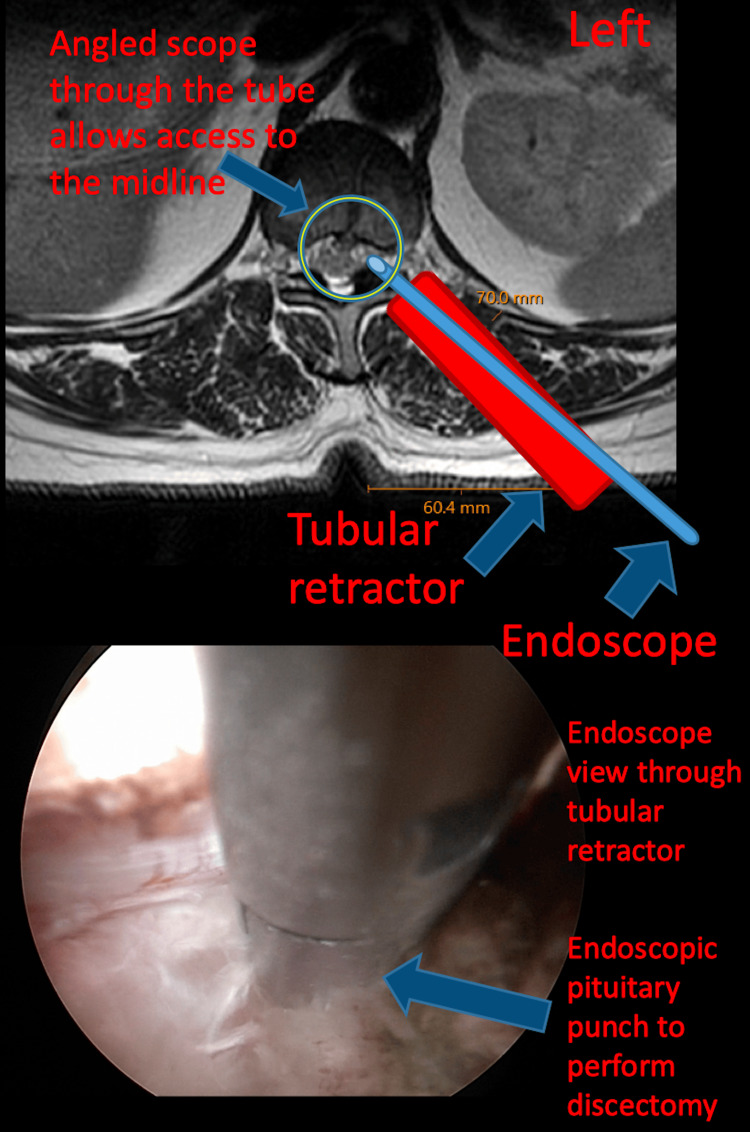
Viewpoint of the endoscope Angled view of the endoscope allowed an endoscopic pituitary punch to perform the ventral discectomy without significant manipulation of the thecal sac.

The microscope was removed and the spine endoscope was placed through the tubular retractor. The angled view of the endoscope allowed an endoscopic pituitary punch to perform the ventral discectomy without significant manipulation of the thecal sac (Figure 3).

**Figure 4:**
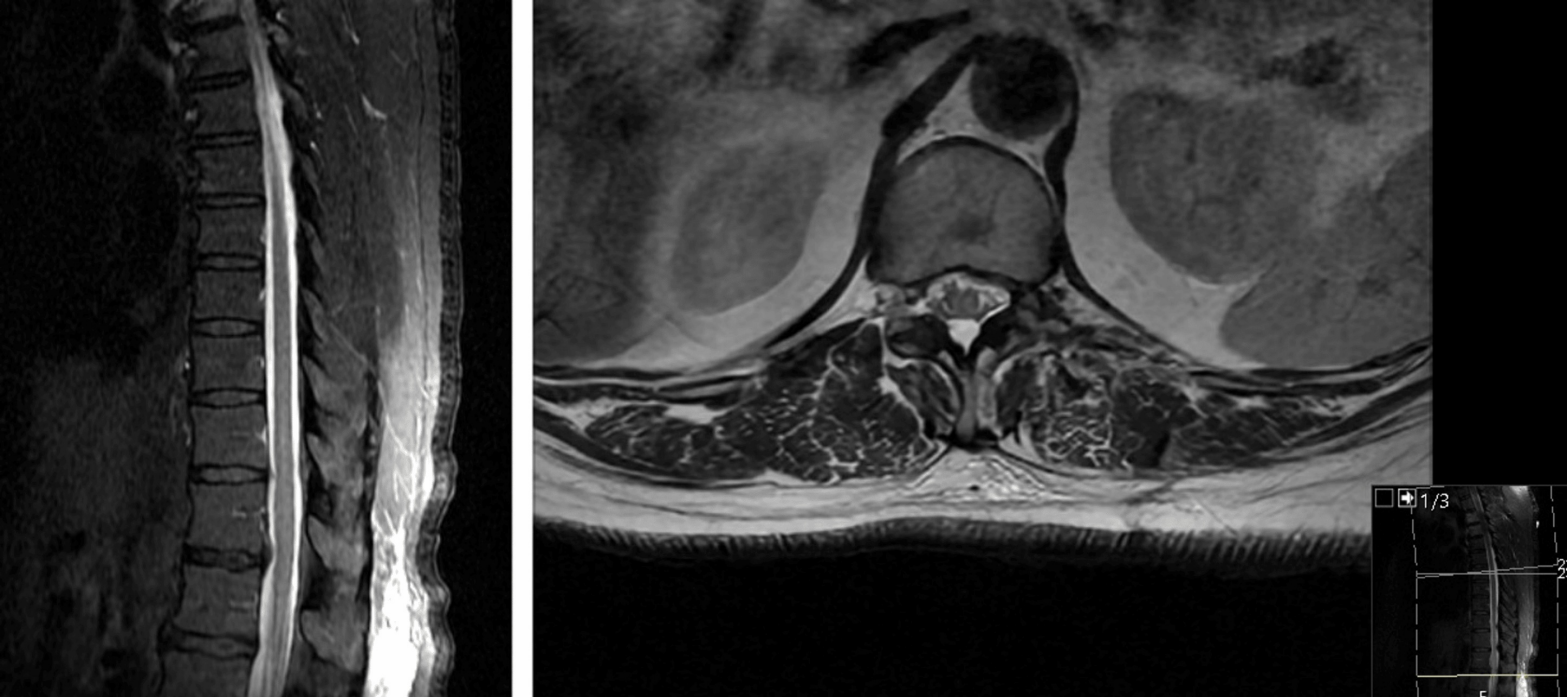
Postoperative imaging Postoperative magnetic resonance imaging (MRI) in the sagittal (A) and axial (B) planes demonstrating stable postoperative changes.

The patient was discharged on postoperative day 1 and demonstrated improving balance at the three-week postoperative visit. She denied thoracic pain and radicular symptoms at this postoperative visit as well. Her thoracic myelopathy was improving and her lumbar symptoms improved. Follow-up MRI revealed stable postoperative changes (Figure 4).

## Discussion

TDHs have an estimated incidence as low as one in one million and have a variable clinical presentation [6]. The presentation can appear like diseases of other organ systems, such as back pain radiating to the chest like cardiac disease, and can be asymptomatic in up to 37% of patients [6]. The surgical approaches to treat TDH include posterior, posterolateral, lateral, transthoracic, and thoracoscopic [6]. Anterior and lateral approaches are favorable for reaching the intervertebral disc and vertebral body, but the lungs, heart, and great vessels can be endangered [6]. For this reason, posterior approaches are considered safer but often necessitate a large incision and a high quantity of bone removal, which can increase blood loss, worsen paraspinal pain, and cause spine instability [6]. Evidence continues to emerge that endoscopic approaches for thoracic spine surgery have many advantages. Some of the benefits include less tissue dissection, blood loss, and epidural fibrosis [5]. Patients who benefit from the minimally invasive approach include the elderly and immunocompromised populations [5]. Microendoscopic and microscopic discectomy work well for TDHs that are soft, lateral, and small [7].

Despite the benefits of endoscopic spine surgery, the technique is challenging. As described by Lewandrowski et al. [8], 15-80 cases are required to master the learning curve for lumbar endoscopic spine surgery [8,9]. The authors discuss the transition from an open approach to other minimally invasive techniques [8]. Using a tubular retractor and microscope involves a viewpoint that surgeons are comfortable adopting from an open approach [8]. The endoscopic viewpoint is different because of the greater detail in pathological site visualization [8]. The surgeon must look away from the surgical field and at the video screen [8]. The endoscopic instruments are longer and require enhanced hand-eye coordination [8,10]. The endoscopic approach involves decreased tactile feedback in comparison to open approaches and a two-dimensional visualization lacking the same level of depth perception as the microscope [6]. It is recommended to begin with more straightforward endoscopic cases and avoid complex herniations and the thoracolumbar region [8]. One of the great challenges of the thoracic region involves the blood supply of the thoracic cord, especially the upper thoracic region with its watershed blood supply that when disrupted can lead to ischemic complications [6]. The thoracic spinal cord does not tolerate retraction to the same extent as other regions. Accessing the disc without interfering with vital structures in the surrounding region is difficult [7].

Several methods have been developed to master the learning curve of endoscopic approaches. Many surgeons learn through conference workshops [8]. Some authors suggest that surgeons learn under an “endoscopic master spine surgeon” [9]. Another challenge is the variation in endoscopic techniques. Phan et al. [11] performed a systematic review comparing the different types of endoscopic approaches and open surgery [11]. The authors found no difference in complications between endoscopic and open approaches [11], demonstrating that endoscopic surgery is worth the time invested in training.

For our patient with T11-T12 TDH and myelopathy, we performed a combined microscopic and endoscopic discectomy. The exposure and bone removal were performed under the microscope. The discectomy was performed with the endoscope. We advocate for this combined approach as a training modality for spine surgeons eager to learn endoscopic techniques. The microscopic portion allows surgeons to use techniques with which they are already familiar. Powered burrs and drills are used under the microscope. These tools have a higher rate of complications like incidental durotomies but are less monotonous than using manual tools [8]. For beginning endoscopic surgeons, it is recommended to use the manual tools [8]. For beginners using the combined microscopic and endoscopic approach, powered tools can be used with the microscope. As described by Snyder et al. [7] in their review, Jho [12,13] published a transpedicular minimally invasive technique of thoracic disc resection involving a tubular retractor, microscope, and endoscope [7,12,13]. The microscope was utilized for drilling off the facet and part of the pedicle [7,12,13]. The endoscope allowed viewing of the ventral thoracic cord, and the herniated disc pieces were extracted from within the intervertebral space [7,12,13]. This approach may be done with fewer incisions and a more favorable morbidity profile than thoracoscopic techniques [7,12,13]. Some surgeons use a posterolateral tubular approach, but the limitation is that the midline cannot be directly visualized, and to overcome the limitations of indirect approaches we added the use of the endoscope. It is difficult to obtain a favorable angle of attack for the disc unless a complete facetectomy is performed. In this case, instability and instrumented fusion are brought into the equation. The combination of microscopic and endoscopic techniques serves as a bridge to mastering more complex endoscopic procedures.

## Conclusions

There are several open and minimally invasive approaches to treat TDHs. Endoscopic thoracic discectomy offers many benefits but also has a difficult learning curve. As more surgeons adopt endoscopic techniques, innovative training modalities are required. We propose a combined microscopic and endoscopic approach that allows surgeons already comfortable with microscopic surgery to improve in endoscopic technique. In particular, we demonstrate that a tubular retractor with the use of a microscope can be implemented to perform the soft tissue dissection and bone removal parts of the case. The endoscope can then be used for the discectomy part of the case. Spine surgeons who already have experience with minimally invasive techniques such as the use of the microscope will likely feel more comfortable with these portions of the case, and this allows them to slowly implement endoscopic techniques into their practice. Once the surgeon performs enough combined microscopic and endoscopic cases, they can begin to take on full endoscopic cases.
